# A Novel Approach to Primary Cell Culture for *Octopus vulgaris* Neurons

**DOI:** 10.3389/fphys.2018.00220

**Published:** 2018-04-03

**Authors:** Valeria Maselli, Fenglian Xu, Naweed I. Syed, Gianluca Polese, Anna Di Cosmo

**Affiliations:** ^1^Department of Biology, University of Naples Federico II, Napoli, Italy; ^2^Department of Biology, Saint Louis University, Saint Louis, MO, United States; ^3^Department of Cell Biology and Anatomy, Cumming School of Medicine, University of Calgary, Calgary, AB, Canada

**Keywords:** primary neuron cell culture, *Octopus vulgaris*, cephalopods, marine invertebrates, central nervous system, vertical-superior frontal system, optic lobes, axon regeneration

## Abstract

*Octopus vulgaris* is a unique model system for studying complex behaviors in animals. It has a large and centralized nervous system made up of lobes that are involved in controlling various sophisticated behaviors. As such, it may be considered as a model organism for untangling the neuronal mechanisms underlying behaviors—including learning and memory. However, despite considerable efforts, Octopus lags behind its other counterparts vis-à-vis its utility in deciphering the cellular, molecular and synaptic mechanisms underlying various behaviors. This study represents a novel approach designed to establish a neuronal cell culture protocol that makes this species amenable to further exploitation as a model system. Here we developed a protocol that enables dissociation of neurons from two specific Octopus' brain regions, the vertical-superior frontal system and the optic lobes, which are involved in memory, learning, sensory integration and adult neurogenesis. In particular, cells dissociated with enzyme papain and cultured on Poly-D-Lysine-coated dishes with L15-medium and fetal bovine serum yielded high neuronal survival, axon growth, and re-growth after injury. This model was also explored to define optimal culture conditions and to demonstrate the regenerative capabilities of adult Octopus neurons after axotomy. This study thus further underscores the importance of Octopus neurons as a model system for deciphering fundamental molecular and cellular mechanism of complex brain function and underlying behaviors.

## Introduction

Due mainly to the complexity of both vertebrate and invertebrate nervous systems, our understanding of the fundamental mechanisms ranging from simple reflexes to complex motor patterns and learning and memory has remained limited. Specifically, it is often difficult to decipher how individual or networks of neurons generate patterned activity underlying any given rhythmic behavior or even invoke mechanisms underlying cognition and learning and memory. *In vitro* cell culture technique represents an important tool in a variety of studies with many applications ranging from biological to medical sciences. *In vitro* cultured cells enable a reductionist approach, which is used as alternative tools instead of animal experimentation, for biotechnological applications and pathological investigations. Such studies have played pivotal roles in deciphering mechanisms of cellular excitability to rhythmogenesis at a resolution not approachable in the intact brain (Schmold and Syed, 2012).

*In vitro* studies on neurons derived from the nervous system of vertebrates such as the chick (Hammarback et al., 1985), frog (Lohof et al., 1992), mouse (Lumsden and Davies, 1986), and rat (Tessier-Lavigne et al., 1988) have been essential to our understanding of neuronal cell biology and the molecular mechanisms underlying chemotropic guidance of growing axons and network (Gordon et al., 2013; Zhang and Hu, 2013; Eberwine et al., 2014; Mergenthaler et al., 2014; Bardy et al., 2015; Gawad et al., 2016). Alternatively, invertebrates comprise more than 95% of the animal species (Rinkevich, 1999) and may be considered a major source for cell culture applications. In fact, attempts to maintain and grow invertebrate cells *in vitro* were made quite early in the history of tissue culture, nearly 100 years ago (Gomot, 1971; Rannou, 1971). Currently, there have been more than 200 cell lines established from tissues of insects and ticks(Bayne, 1998), in particular *Drosophila melanogaster* (Gonzalez et al., 2011) and *Caenorhabditis elegans* (Christensen et al., 2002; Strange and Morrison, 2006).

In marine invertebrates, there are only limited primary cell cultures/cell lines developed from a few species within six invertebrate phyla (Porifera, Cnidaria, Crustacea, Mollusca, Echinodermata, Urochordata) out of more than 30 invertebrate phyla available, even though they represent a rich source of cell and tissue types and they significantly differ from one group to another (Rinkevich, 1999). Molluscs are probably the most intensively studied group of marine invertebrates as it comes to cell culture techniques (Syed et al., 1999; Schmold and Syed, 2012). During the last 20 years, a variety of organs and cells from molluscs have been cultured, including epithelial cells from embryos, gills and mantles (Cornet, 1995), nervous system (Berdan et al., 1990; Tamse et al., 1995), digestive glands (Odintsova et al., 1994), cardiac muscles (Kleinschuster et al., 1996), giant fiber lobe neurons of the squid (Gilly et al., 1990), and the hematopoietic systems (Davids and Yoshino, 1998; Troncone et al., 2015). In particular, primary cultures of neurons from molluscs have been extensively used for studies on neural growth, axon pathfinding, synapse formation, and nerve regeneration (Syed et al., 1990). Primary cultures of several types of crustacean neurons have also been developed previously (Toullec, 1999), among which the most developed culture conditions are for olfactory sensory neurons and stomatogastric neurons (Graf and Cooke, 1990; Fadool et al., 1991; Zhao et al., 2009).

Our major objective was to develop a neuron cell culture protocol since there were no such techniques available for octopus neurons and all previous attempts were unsuccessful.

Inspired by cell culture work on other invertebrates including molluscs and crustaceans, we set out here to develop a protocol for primary culture of neurons from the nervous system of the cephalopod *Octopus vulgaris*—a powerful model system, that could serve us well in deciphering the behavioral, cellular, and molecular mechanisms at the basis of brain function.

*Octopus vulgaris* lives an active life, it has a closed vascular system, a vertebrate like blood-brain barrier, possesses complex and centralized nervous system, exhibiting sophisticated behaviors (Young, 1971; Nixon and Young, 2003). Its “intelligence” coupled to some intriguing characteristics of its brain, such as the presence of nervous districts comparable to vertebrates' specific areas, and the absence of myelination in neurons indicated that octopus may be an excellent animal model characterized by a separate evolutionary lineage, which achieved many neuronal complexities independently (Hochner, 2010; De Lisa et al., 2012a,b).

Despite extensive studies and the advanced knowledge that we have gained vis-à-vis octopus's high cognitive abilities *in vivo*, learning and memory skills (Byrne et al., 2006; Kuba et al., 2006a,b, 2010; Gutnick et al., 2011; Tramacere et al., 2013; Richter et al., 2015, 2016) and the recent sequencing of *O. bimaculoides* genome and *O. bimaculoides* and *O. vulgaris* transcriptomes (Albertin et al., 2012; Zhang et al., 2012; Liscovitch-Brauer et al., 2017), almost nothing is known about the cellular mechanisms underlying behaviors at the associative neuronal network level, due mainly to the lack of various cell culture techniques, except for white body cells in *O. vulgaris* (Necco and Martin, 1963) and the stellate ganglion of *O. rubescens* (Gilly et al., 1997).

In view of the above need and motivation, here we developed a novel technique to isolate and culture primary neurons from the *O. vulgaris* brain, accomplishing the following aims: (1) developed a protocol to obtain dissociated neurons from two specific brain areas, the vertical-superior frontal system (VSFS) and the optic lobes (OL), two brain regions are involved in memory, learning, sensory integration and adult neurogenesis in *Octopus vulgaris* (Bertapelle et al., 2017); (2) compared different cell culture coating reagents and culture medium supplements for cell adherence, survival, and growth; (3) conducted immunocytochemistry analysis of neuronal markers; lastly (4) demonstrated, for the first time, that *in vitro* VSFS neurons exhibit robust regenerative behavior after axotomy and cell isolation.

## Methods

### Animals

Specimen of *O. vulgaris* (*n* = 3 male, weight ~400 g), collected in Bay of Naples, were maintained in aquarium tanks (Polese et al., 2014; Di Cosmo et al., 2015). This study was carried out in accordance with the recommendations of European Directive 2010/63 EU L276, the Italian DL. 4 /03/ 2014, n. 26 and the ethical principles of Reduction, Refinement and Replacement (protocol n. 0124283-08/11/2012). Octopuses were anesthetized with isoflurane insufflation (Polese et al., 2014) and brains were dissected under sterile conditions. The protocol was approved by the “Centro Servizi Veterinari” of University of Naples Federico II and Ministero della Salute, Ufficio Tutela del Benessere Animale (Prot. 608/2016-PR).

### Hemolymph collection

When octopuses were completely relaxed, the ventral mantle was partially folded backwards to expose the branchial hearts for blood sampling (Malham et al., 1998). The hemolymph was collected using a 2.5 ml sterile syringe with 30G needle. 20 ml of hemolymph was typically obtained and was immediately diluted in an equal volume of marine anticoagulant solution composed of 0.1 M Glucose, 15 mM trisodium citrate, 13 mM citric acid, 10 mM ethylene diaminetetraacetic acid, 0.45 M NaCl, at pH 7.0 and 1,000 mOsm (Barcia et al., 1999). The hemolymph solution was centrifuged at room temperature (21–22°C) for at least 10 min at 2,000 RPM to pellet hemocyte. The supernatant was sterilized through a 0.22 μm pore filter and stored at −20°C.

### Neuronal cell culture

The central nervous system was removed and the vertical-superior frontal system mass (VSFS) and optic lobes (OL) were dissected. They were then separately cut into small pieces with a scalpel and incubated with 1 mg/ml papain enzyme for 30 min at room temperature. For comparison, tissues were also exposed to first collagenase P (4 mg/ml) in L15-supplemented artificial sea water (L15-ASW, containing 400 mM NaCl, 10 mM KCl, 15 mM HEPES, and Phenol Red, pH 7.8) for 30 min at room temperature (21–22°C) and then with 1 mg/ml trypsin in ASW for 20 min at room temperature (all chemicals and enzymes are from Sigma-Aldrich, St. Louis, MO, USA). VSFS and OL were extensively washed in Leibovitz-15 medium (ThermoFisher Scientific, Waltham, MA, USA) to stop the enzyme function. Tissues were triturated with fire polished glass pipette as well as 1 ml and 0.2 ml pipette tips to yield single cells for 2–5 min or until no cell clusters were visable. After VSFS and OL cell dissociation, cells were diluted to an appropriate density in culture media and plated on glass coverslips which were pre-coated with Poly – L - lysine (100 μg/ml, PLL, Sigma Aldrich) or Poly – D - lysine (100 μg/ml, PDL, Sigma Aldrich) in sterile water. Cells were allowed to attach to the coating surface for 30 min prior to the addition of 2 ml of the Leibovitz-15 medium and cells were maintained in an incubator at saturation humidity at 18°C. For comparison study, additive components to the culture medium including either Fetal Bovine Serum (at 4%, FBS) or Hemolymph (at 10%, HEMO) were applied after plating and maintained through subsequent feeding with media. Half of the culture medium was substituted on the third day.

### Trypan blue cell viability assay

Trypan Blue is a staining dye recommended for cell viability evaluation. The medium was gently removed from the dish and Trypan blue (0.04%) in Penicillin-Streptomycin solution (Pen/Strep 1%) was added to the culture for 10 min. The solution was then removed and the culture was washed two times with Pen/Strep solution. The live (non-stained) and dead (Trypan blue-stained) cells were counted under the inverted phase contrast microscopy, and the cell viability percentage was calculated.

### Immunocytochemistry and neuronal regeneration study

Neurons, cultured for 4 days in L15-medium plus 10% HEMO in PLL-coated dishes, were fixed for 20 min with 4% Paraformaldehide in 1X PBS, washed 3 times with 1X PBS, and incubated with blocking solution containing 0.1% Triton-X 100 and 5% Goat Serum in 1X PBS for 1 h at room temperature. Cells were then incubated for 1 h with mouse polyclonal Anti-β III Tubulin antibody (1:500, abcam) and rabbit polyclonal poli (ADP-ribose) polymerase (PARP1) primary antibody (De Lisa et al., 2012a) (1:500, Santa Cruz Biotechnology Inc.) in blocking solution overnight at 4°C. After washing off the primary antibodies with 1X PBS for three times, cells were incubated in dark for 1 h with incubation medium containing secondary antibodies, the FITC-conjugated goat anti-mouse IgG (1:200, Thermo Fisher Scientific) and Rhodamine conjugated goat anti-rabbit IgG (1:200, Thermo Fisher Scientific). Cells were washed three times with 1X PBS and coverslips were mounted with mounting media containing 5% Glycerol and 0.2% DAPI. Fluorescent images were acquired using an A1R MP microscope under a CFI Plan Fluor 20X/0.75 MI objective with NIS Elements v4.13.00 software (Nikon). Fluorophores were excited with 488 nm and 561 nm lasers and emissions collected through 525/50 and 595/50 filter cubes.

In order to test the effectiveness of optimal cell culture conditions for VSFS cells under our culture condition, a neurite transection of a selected neuron was performed on an inverted microscope (Nikon Ti Eclipse E400) using the sharp tip of a pulled glass pipette mounted on a micromanipulator. The neurite re-growth was monitored using the same inverted microscopy for 0–3 h.

### Parameters for the evaluation of cell culture conditions

Phase contrast images of cells under different culture conditions were taken using an inverted microscope (60X magnification, Nikon Ti Eclipse E400). The number of adherent cells with or without neuritic processes in 10 fields of view for each treatment was counted and measured using imageJ software. Neurite exhibiting at least half of the cell body length was counted as a neuronal process. Specifically, the “NeuronJ” plug-in (version 1.4.3) of NIH-ImageJ software (version 1.50 Meijering et al., 2004) was used to quantify the average neurite length of each neuron at different time points (day 1 and day 4). Eight-bit grayscale images of neurons with identifiable neurites were loaded into the software and calibrated according to the image magnification. The average length of the neurites was obtained by manually tracing the length of all neurites from one single neuron's cell body and divided by the total number of neurites per neuron. The lengths and the total number of neurites were averaged across all neurons in each treatment.

Boxplots resulted from 10 images per treatment for the following four parameters: the number of cells attached, the number of cells exhibited neurites outgrowth, the number of neurites, and the neuritic length. The top and bottom of the boxes mark the 25th and 75th centiles and the inner line marks the median value; 25% of the data above the 75th centile and 25% of the data below the 25th centile are marked as “whiskers” limited by the maximum or minimum values. Outliers are displayed as points.

### Data analysis and significance test

The experimental data were collected from four separate experiments with the examination of different culture conditions. Statistical analyses were performed with (R Developmental Core Team, 2011). Differences in distribution were tested using Kruskal-Wallis test, the difference was deemed to be significant at the level of *p* < 0.05.

## Results

Our dissociation approach yielded different cell types (Figure S1) which were selected on the basis of the morphology and dimension. In particular, we selected amacrine cells of 10 μm diameter from VSFS and 10 μm diameter from OL.

### The effects of dissociation enzymes, coating regents, and culture media on neuronal culture efficacy

**Figure 1:**
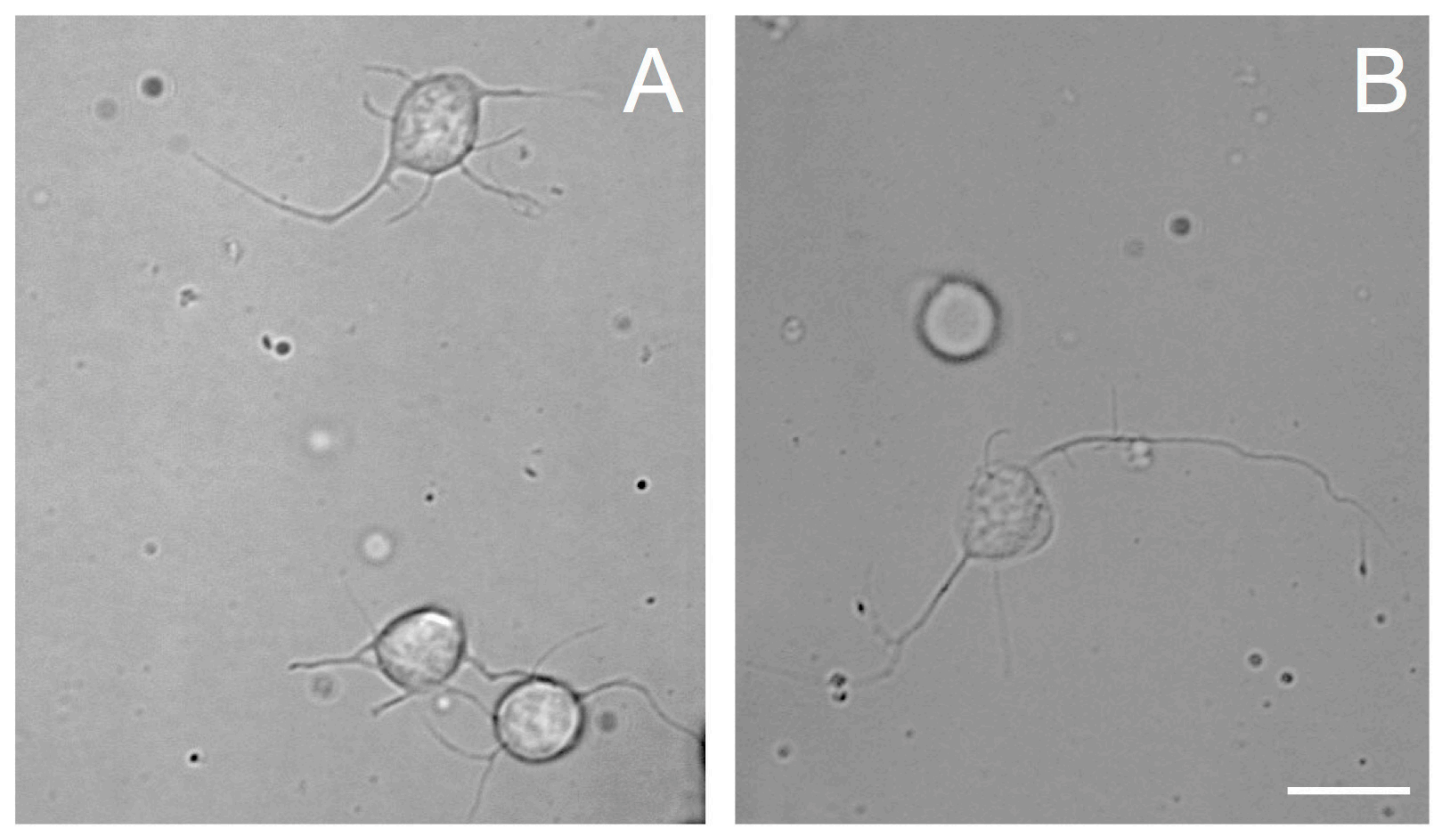
Phase contrast images of cells from VSFS dissociated with papain **(A)** or collagenase **(B)** enzyme, plated on PDL coated glass coverslips and cultured in L15 medium; white scale bar indicates 10 μm.

**Figure 2:**
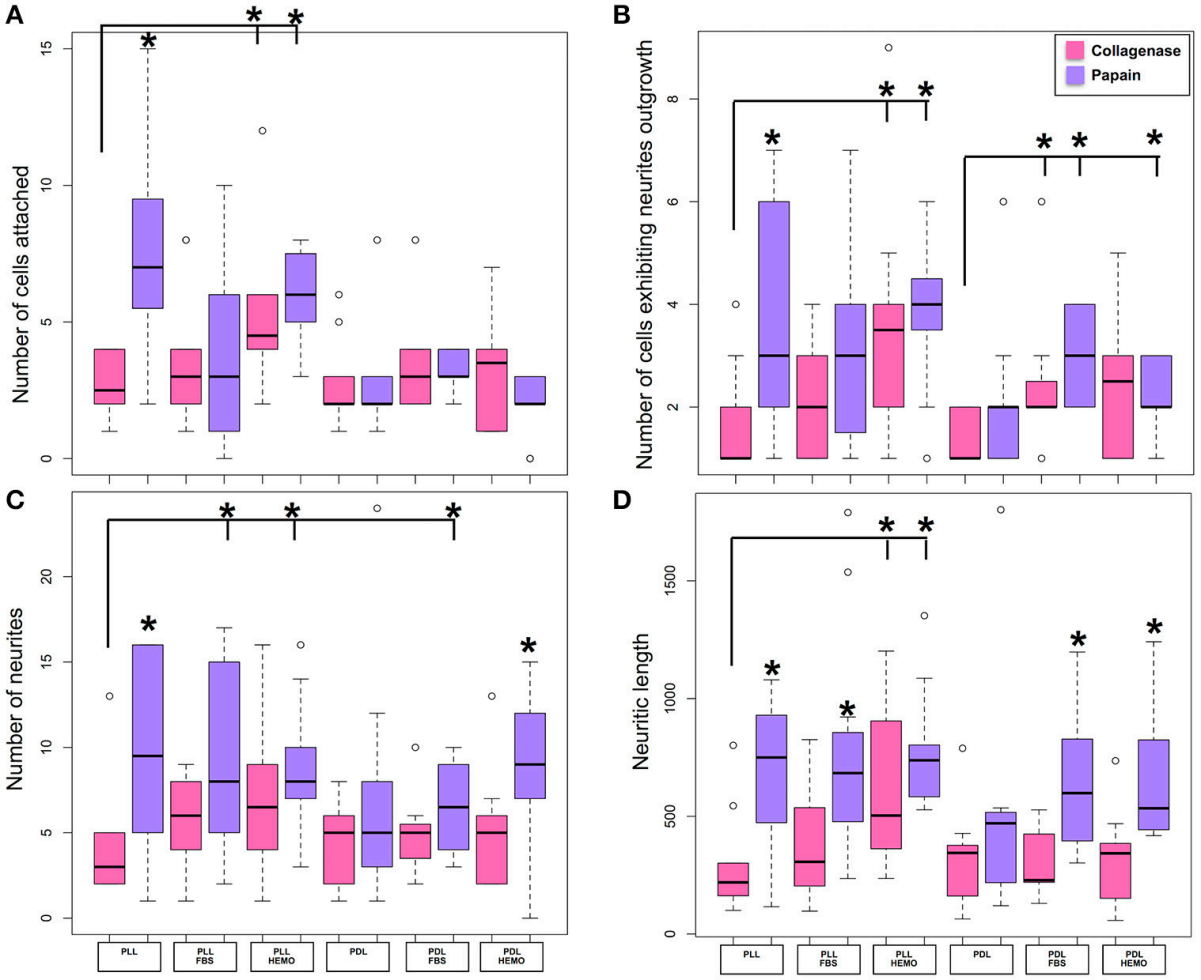
Number of cells attached and exhibited neurites outgrowth and number of neurites and neuritic length of VSFS cells when dissociated with collagenase (pink) and papain (purple), plated on PLL and PDL coated glass coverslips, and cultured in L15 medium with the addition of FBS or HEMO; ^*^p < 0.05.

To establish an optimal cell culture protocol, we sought to test the efficiency of commonly used dissociation enzymes, papain or collagenase in combination with trypsin, on VSFS cells plated on either PLL or PDL pre-coated dishes in L15 medium with the addition of FBS 4% or HEMO 10% (Figures 1, 2). After 1 day of cell culture, phase contrast images were taken and four parameters were evaluated: the number of cells attached and those that exhibited neurites outgrowth, and the number of neurites and neuritic length (Figures 1, 2).

The choice of these enzymes are based on the fact that Octopus nervous system is rich in both collagen based connective tissues and adhesive proteins surrounding the neurons (Kier and Stella, 2007). Interestingly, we found that both papain and collagenase-trypsin were equally potent in breaking down the octopus VSFS neural tissue into single neurons (Figure 1). These neurons exhibited round shaped cell bodies, whose diameters ranged between 5 and 10 μm. After 1 day in culture, the majority of cells, in all culture conditions, sent out fine and branched processes (Figure 1). Although both enzymes showed strong dissociation efficiency, interestingly, our statistical data revealed that neurons dissociated with papain demonstrated better performances than those dissociated with collagenase, in all coating and culture conditions (Figure 2 and Table 1).

**Table 1:** Mean ± standard error of four quantitative parameters for evaluating the effectiveness of cell culture conditions with collagenase (C) or papain (P) enzymes.

**Parameters evaluated**	**Number of cells attached**		**Number of cells exhibiting neurites outgrowth**		**Numbers of neurites**		**Neuritic length**	
**Enzyme**	**C**	**P**	**C**	**P**	**C**	**P**	**C**	**P**
PLL	2.50 ± 0.40	7.00 ± 1.10^*^	1.00 ± 0.34	2.50 ± 0.67^*^	3.00 ± 1.06	7.50 ± 1.78^*^	219.02 ± 68.22	742.85 ± 113.98^*^
PLL+FBS	3.00 ± 0.78	3.00 ± 0.89	2.00 ± 0.31	2.00 ± 0.58	6.00 ± 0.83	7.00 ± 1.75^*^	306.45 ± 79.18	657.40 ± 148.23^*^
PLL+HEMO	4.50 ± 0.90^*^	6.00 ± 0.48^*^	3.50 ± 0.72^*^	4.00 ± 0.42^*^	6.50 ± 1.45	8.00 ± 1.13^*^	503.07 ± 111.20^*^	737.58 ± 75.81^*^
PDL	2.00 ± 0.58	2.00 ± 0.63	1.00 ± 0.17	2.00 ± 0.48	5.00 ± 0.91	5.00 ± 2.16	344.34 ± 74.57	470.02 ± 150.10
PDL+FBS	3.00 ± 0.81	3.00 ± 0.25	2.00 ± 0.61^*^	3.00 ± 0.26^*^	5.00 ± 0.98	6.50 ± 0.76	228.62 ± 59.32	598.20 ± 98.37^*^
PDL+HEMO	3.50 ± 0.67	2.00 ± 0.35	2.50 ± 0.50	2.00 ± 0.36^*^	5.00 ± 1.05	8.00 ± 1.70^*^	342.60 ± 60.95	473.80 ± 123.05^*^

Statistical differences are in bold and asterisks indicate positive statistical significance (^*^).

Specifically, neurons dissociated with papain enzyme exhibited a significantly higher degree of attachment (reflected by a higher cell count per image area of 12,000 μm^2^) and more extensive neurite outgrowth (reflected by a larger number of cells developing neurites, more neuritic branches, and longer neuritic processes). Note that papain culture showed less cellular debris as compared to collagenase culture and contained more neurons, which had bright, phase contrast membrane boundaries (Figure 1). Furthermore, our results demonstrate that cells cultured on PLL-coated coverslips (the left panel of Figures 2A–D) performed significantly better than neurons grown on PDL-coated surfaces (right panel of Figures 2A–D) in all parameters measured (Table 1). These data indicate that papain dissociation in couple with PLL-coating is a good combination procedure for promoting neuronal attachment and subsequent growth during *in vitro* culture of VSFS neurons.

Because neuronal endogenous factors such as HEMO and exogenous molecules like FBS, could provide growth factors essential for cell survival, migration, differentiation, as well as neurite initiation, process extension, and nerve regenerations both *in vivo* and *in vitro* (Gordon et al., 2013), we tested the effects of these factors by including them in the L-15 culture media. Our results demonstrate that in PLL coated dishes, neurons exposed to HEMO and FBS exhibited a similar cell count and growth when compared with neurons in the absence of these growth factors. Interestingly, both HEMO and FBS significantly promoted the growth of neurons (more cells have growth and each neuron has more branches and longer neurites) cultured in PDL-coated dishes as shown in Figures 2B–D, despite the fact that the number of cells attached to the substrate was not affected (Figure 2A). It is important to note that overall the cells cultured in PLL dishes in the absence or presence of HEMO or FBS (the left six columns) exhibited similar or better cell count and growth as compared to neurons cultured in PDL dishes with the addition of HEMO or FBS (the right four columns). These results indicate that the coating agent PLL itself is more effective for octopus cell culture and the endogenous or exogenous factors are beneficial to neurons that are cultured on PDL-coated substrates. In summary, these data indicate that the optimal cell culture of octopus VSFS neurons could be achieved by culturing cells on PLL subtract coated dishes either with or without HEMO or FBS or on PDL-coated dishes with the addition of HEMO or FBS to the media.

### Cell viability and growth of VSFS cells in culture

**Figure 3:**
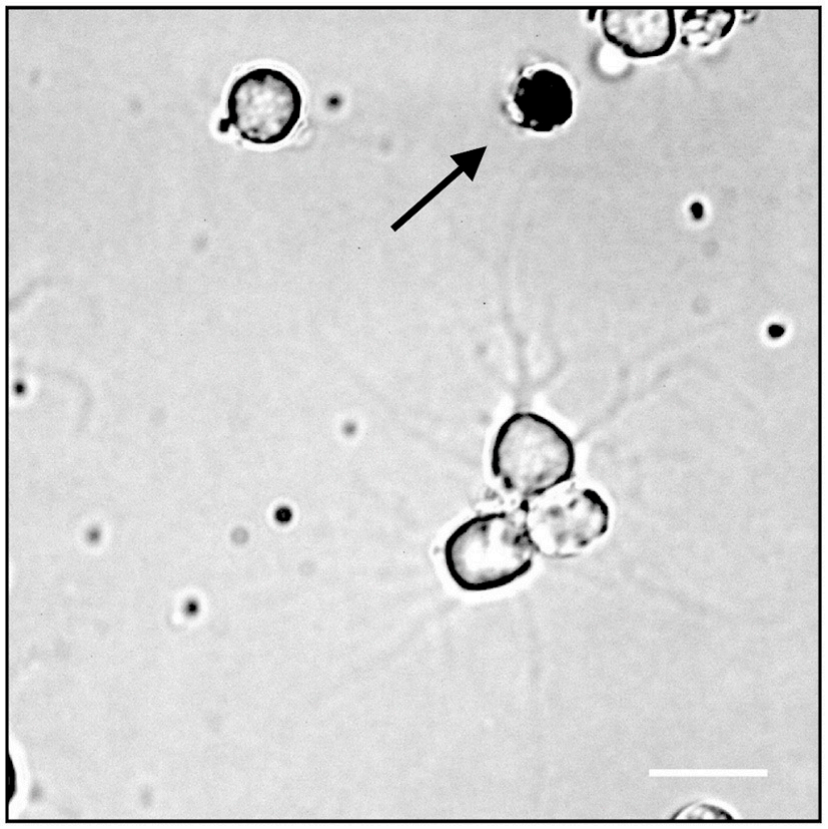
Trypan Blue assay, the arrow indicates a dead neuron cell; white scale bar indicates 10 μm.

To test the cell health condition in our culture, we next performed the live/dead cell assay using the standard Trypan Blue staining method. Cells dissociated with papain were cultured on PLL coated dishes overnight and stained with Trypan blue solution the next day (see section Method). Figure 3 shows that the majority of cells (94.60%, ±1.78) did not show any staining with the blue dye indicating that they were all alive. Note that only very few unhealthy cells were stained with the dye. These data clearly demonstrated that cells under our culture procedure are very healthy.

**Figure 4:**
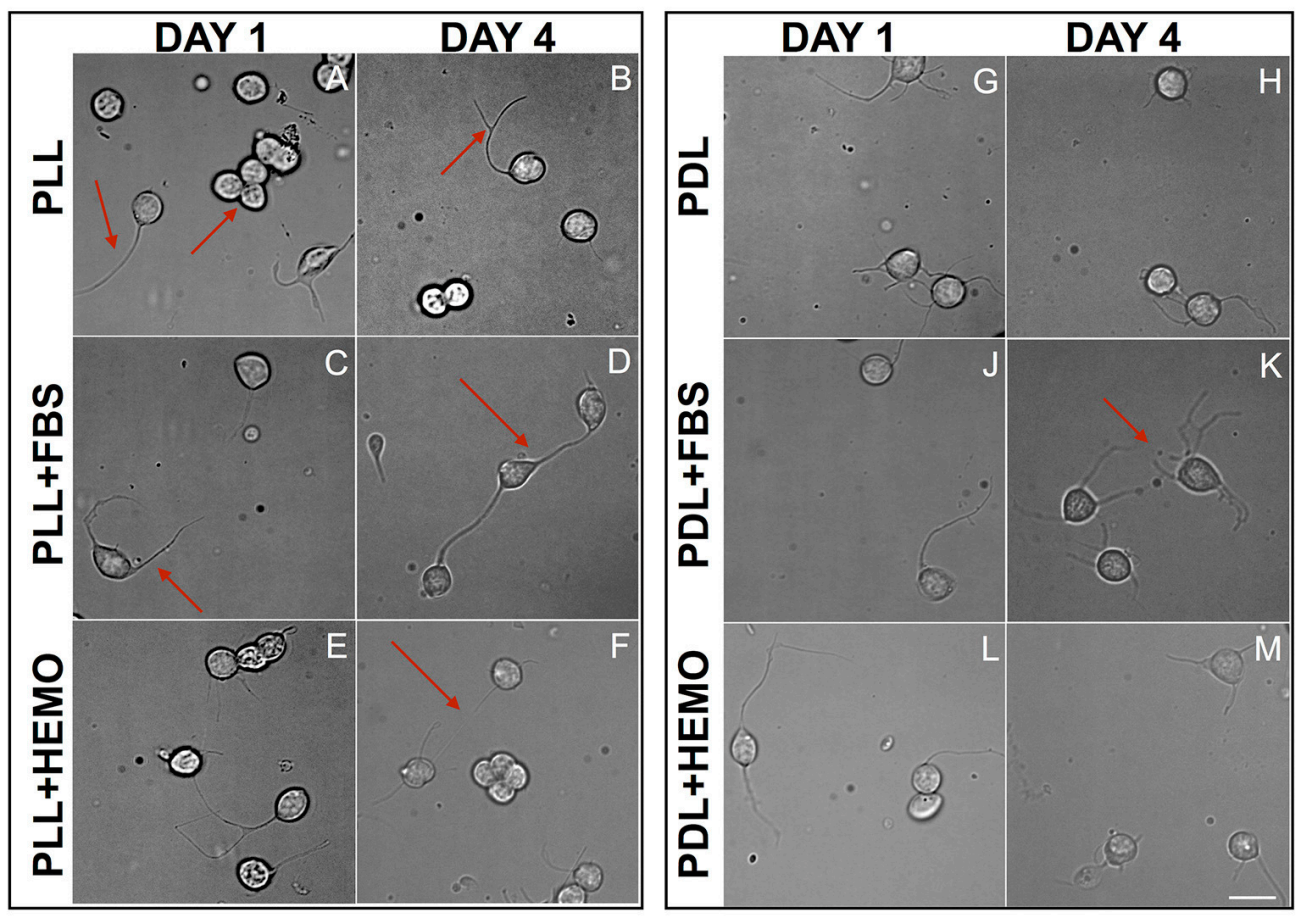
Cells from VSFS plated on PLL **(A–F)** and PDL **(G–M)** coated dishes, cultured for 4 days in L15 medium with the addition of FBS **(C,D,J,K)** or HEMO **(E,F,L,M)**; Red arrows indicate: **(A)** neurite and neurons cluster; **(B)** branched neurite; **(C)** neurite; **(D,F)** axo-somatic contacts; **(K)** neurite networks; white scale bar indicates 10 μm.

To further confirm that these neurons were indeed healthy, we monitored their growth and tested their ability to develop neuritic contacts. Cells treated with papain were plated on PLL or PDL coated dishes cultured in L15 medium with the addition of FBS 4% or HEMO 10% for up to 4 days, images were acquired from cultures in both day 1 and day 4 (Figure 4).

We chose these two-time points as they guaranteed to clearly differentiate among different treatments, and based on our observations that neurons start to show physical contact within 3–4 days. We found that the majority of the cells, in all treatments, send out fine and branched processes from day one of culture as described earlier (Figure 4). Specifically, we observed round shaped cells in PLL coated dishes after the 1st day and some of them spread out either one or two neurites or in clusters (Figure 4A). At the 4th day in culture, cells exhibited round shapes, showing branched neurites and more of these cells developed either “soma-soma” clusters, soma-neurite or neurite-neurite contacts. It is important to note that cells cultured in the presence of FBS in both PLL and PDL coated dishes exhibited more extensive branches and cell contact after 4 days in culture. Our data indicate that neurons continue to grow up nicely and develop physical contacts with each other within 4 days of culture.

Comparing PLL + FBS at 1st and 4th day of culture (Figures 4C,D) we observed respectively cells showing morphology quite similar to that of PLL at 1st day (Figure 4A) and only at the 4th day of culture axo-somatic contacts were observed (Figure 4D).

In PLL + HEMO, the 1st day was dominated by the presence of round cells with few branching neurites (Figure 4E), whereas on the 4th day small clusters and axo-somatic contacts were observed (Figure 4F). In PDL plated culture at 1st and 4th day, neurons exhibited round shapes with many branched neurites involved in axo-somatic and axo-axonic contacts (Figures 4G,H).

In PDL + FBS at 1st day, neurons exhibited long neurites (Figure 4J) and on 4th day (Figure 4K), they formed networks. In PDL + HEMO at 1st day, the round shaped neurons showed long branched neurites (Figure 4L), at 4th day the neurons showed more shorter neurites (Figure 4M).

**Figure 5:**
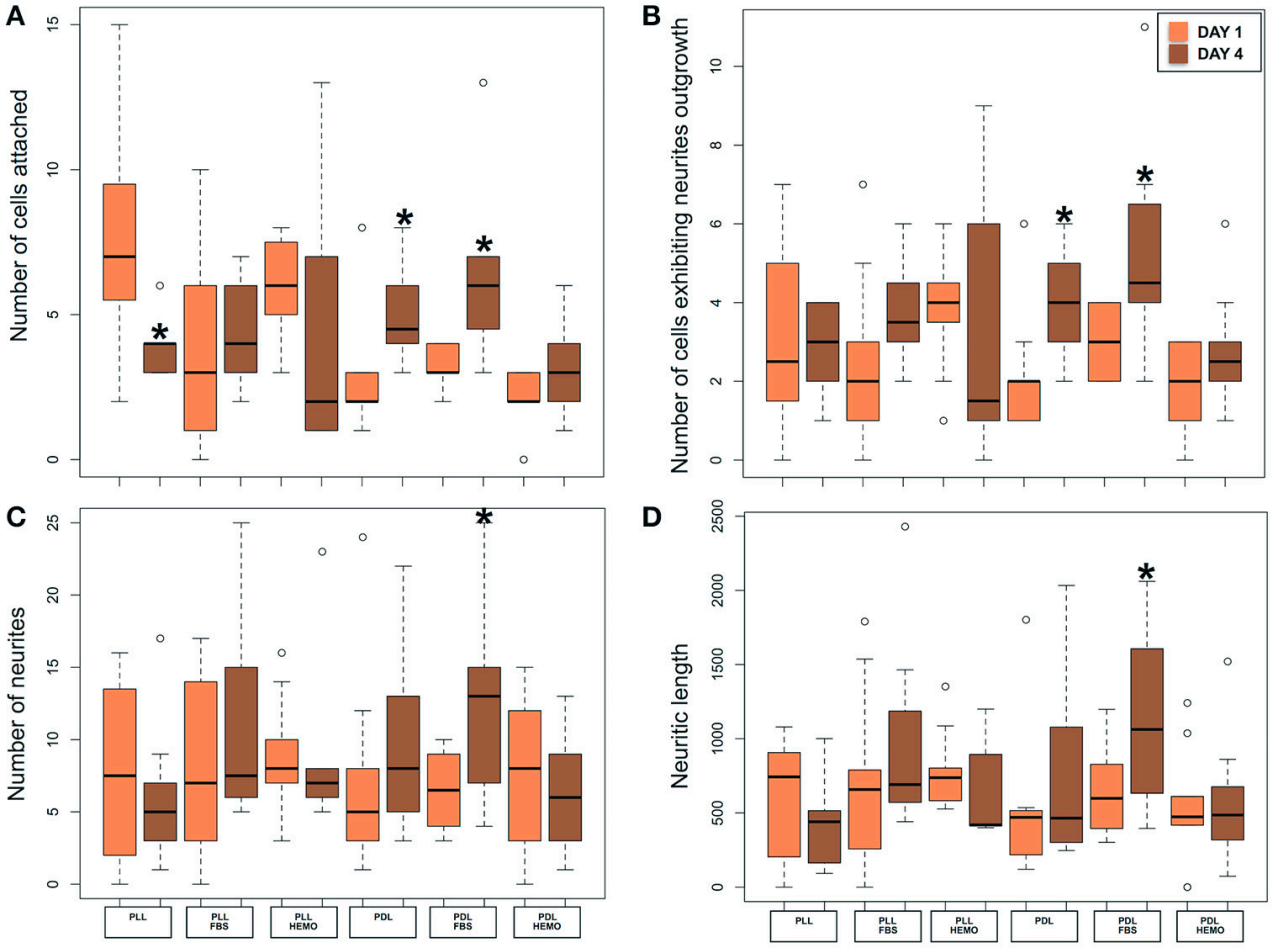
Number of cells attached **(A)** and exhibited neurites outgrowth **(B)**; number of neurites **(C)** and neuritic length **(D)** from VSFS cells in *Octopus vulgaris* plated on PLL and PDL coated dishes, cultured for 4 days in L15 medium with the addition of FBS or HEMO; ^*^p < 0.05.

Our statistical analysis revealed that VSFS neurons cultured on PDL-coated dishes with the addition of FBS, and not HEMO, show significant increase in all parameters evaluated after 4 days in culture compared to neurons at 1 day culture (Figure 5, Table 2). It is important to note that the number of adhered cells and their growth in PLL-coated dishes (with or without the addition of FBS and HEMO) and PDL + HEMO did not show an increase from the 1st to the 4th day of culture, meanwhile in PDL and PDL + FBS exhibited an increase from the 1st to the 4th day of culture (Figure 5). Specifically, a similar trend was observed considering the number of cells with neurites outgrowth from 1st to 4th day of cell culture in PLL, PLL enriched (both FBS and HEMO) and PDL + HEMO, meanwhile in PDL and PDL + FBS demonstrated a positive trend (Figure 5B). The number of neurites increased only in PDL + FBS treatment from the 1st to 4th day of culture (Figure 5C). The length of neurite mirrored the same trend, showing an increase only in PDL + FBS treatment from the 1st to 4th day of culture (Figure 5D).

**Table 2:** Mean ± standard error of four quantitative parameters used to evaluate the effectiveness of VSFS culture condition the 1st day (1) and the 4th day (4).

**Parameters evaluated**	**Number of cells attached**		**Number of cells exhibiting neurites outgrowth**		**Numbers of neurites**		**Neuritic length**	
**DAY**	**1**	**4**	**1**	**4**	**1**	**4**	**1**	**4**
PLL	7.00 ± 1.10	**4.00 ± 0.32−**	2.50 ± 0.67	3.00 ± 0.41	7.50 ± 1.78	5.00 ± 1.59	742.85 ± 113.98	440.75 ± 98.55
PLL+FBS	3.00 ± 0.89	4.00 ± 0.48	2.00 ± 0.58	3.50 ± 0.39	7.00 ± 1.75	7.50 ± 1.84	657.40 ± 148.23	691.11 ± 165.01
PLL+HEMO	6.00 ± 0.48	2.00 ± 1.96	4.00 ± 0.42	1.50 ± 1.45	8.00 ± 1.13	6.50 ± 3.18	737.58 ± 75.81	415.98 ± 173.30
PDL	2.00 ± 0.63	4.50 ± 0.53^*^	2.00 ± 0.48	4.00 ± 0.42^*^	5.00 ± 2.16	8.00 ± 1.84	470.02 ± 150.10	464.91 ± 179.26
PDL+FBS	3.00 ± 0.25	6.00 ± 0.73^*^	3.00 ± 0.26	4.50 ± 0.69^*^	6.50 ± 0.76	13.00 ± 1.93^*^	598.20 ± 98.37	1,062.70 ± 155.01^*^
PDL+HEMO	2.00 ± 0.35	3.00 ± 0.49	2.00 ± 0.36	2.50 ± 0.47	8.00 ± 1.70	6.00 ± 1.26	473.80 ± 123.05	485.88 ± 128.57

In bold statistical differences, asterisks for positive statistical significance (^*^), minus for negative statistical significance (–).

### Optical Lobe cell culture

**Figure 6:**
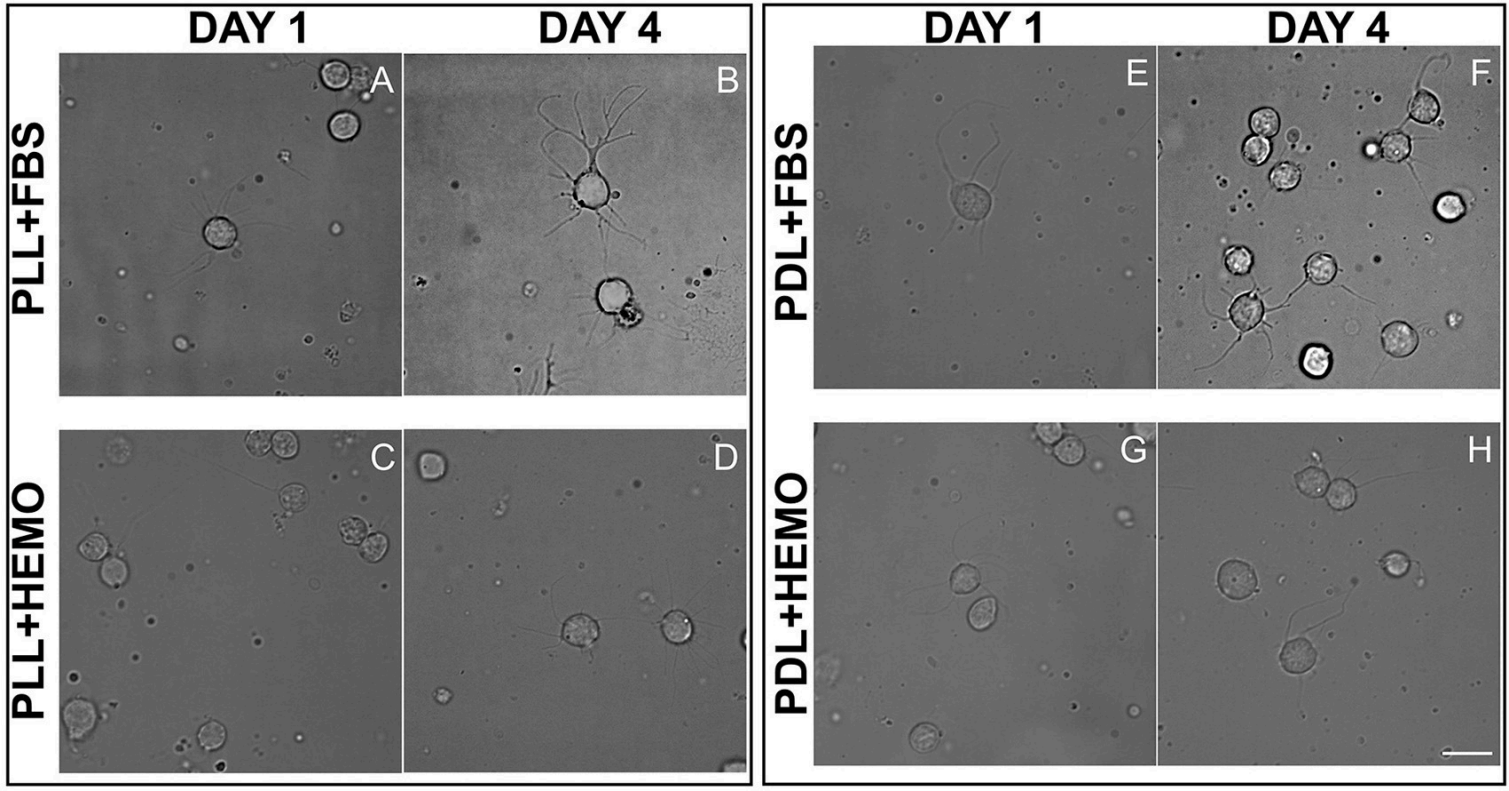
Cells from OL plated on PLL **(A–D)** and PDL **(E–H)** and coated dishes, cultured for 4 days in L15 medium with the addition of FBS **(A,B,E,F)** or HEMO **(C,D,G,H)**; white scale bar indicates 10 μm.

We next tested the effectiveness of our protocol for VSFS neurons on other important lobes of interest, the octopus Optic Lobe (OL, integrative lobes for visual sensations). After OL dissection, we collected a mix of different cell types, neurons from the outer and inner granular layers and neurons from the central part (medulla). We dissociated neurons using papain enzyme and cultured them on PLL or PDL coated dishes in culture medium containing either HEMO or FBS enrichment (Figure 6). We found that all neurons in culture showed similar morphology to that of VSFS neurons, round shaped cell bodies with a cell diameter ranging between 5 and 10 μm and most cells developed fine and branched processes since the first day in culture (Figures 6A–D). Neurons showed an increased number of neurites, branches, and contacts (Figures 6E,F). It is interesting that FBS increased branching and cell-cell contacts when compared with HEMO (Figure 6).

**Figure 7:**
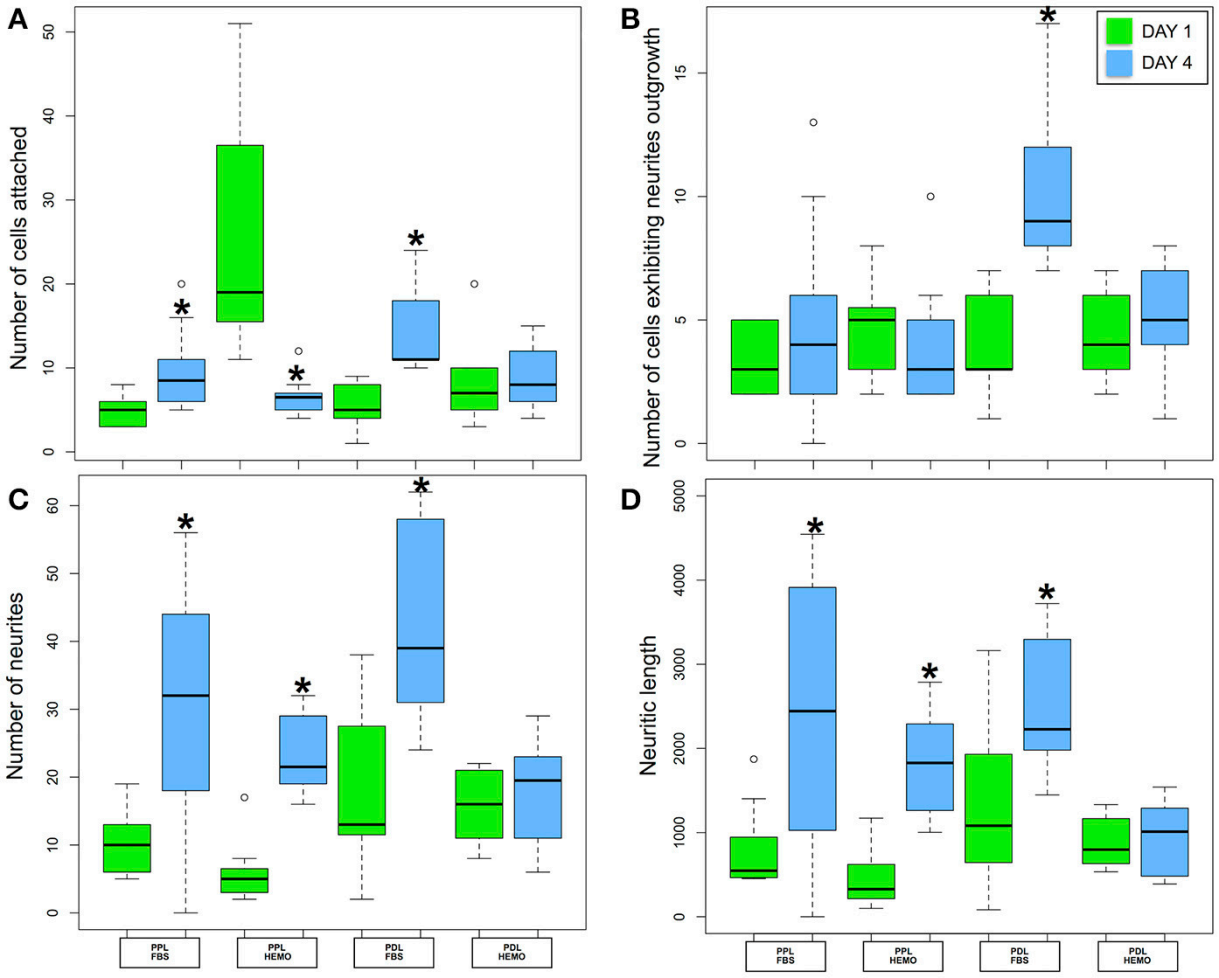
Number of cells attached **(A)** and exhibited neurites outgrowth **(B)**; number of neurites **(C)** and neuritic length **(D)** from OL cells in *Octopus vulgaris* plated on PLL and PDL coated dishes, cultured for 4 days in L15 medium with the addition of FBS or HEMO; ^*^p < 0.05.

Our statistical analysis showed that FBS is more robust as compared to HEMO in promoting neuronal adhesion and neurite outgrowth in 4 days (Figure 7A, Table 3). Specifically, the number of cell count and neurites increased significantly in all treatments from day 1 to day 4 by the addition of FBS. There were no significant differences in cultures of PDL+HEMO between 1st and 4th day of cell culture (Figures 7A,C, Table 3).

**Table 3:** Mean ± standard error of four quantitative parameters used to evaluate the effectiveness of OL culture condition the 1st day (1) and the 4th day (4).

**Parameters evaluated**	**Number of cells attached**		**Number of cells exhibiting neurites outgrowth**		**Numbers of neurites**		**Neuritic length**	
**DAY**	**1**	**4**	**1**	**4**	**1**	**4**	**1**	**4**
PLL+FBS	5.00 ± 0.55	8.50 ± 1.16^*^	3.00 ± 0.40	4.00 ± 0.95	10.00 ± 1.58	32.00 ± 4.66^*^	546.47 ± 152.86	2,443.55 ± 1,239.76^*^
PLL+HEMO	19.00 ± 4.26	**6.50 ± 0.73**−	5.00 ± 0.59	3.00 ± 0.81	5.00 ± 1.26	21.50 ± 1.91^*^	327.12 ± 96.60	1,827.71 ± 194.49^*^
PDL+FBS	5.00 ± 0.82	11.00 ± 2.71^*^	3.00 ± 0.59	9.00 ± 1.81^*^	13.00 ± 3.33	39.00 ± 7.44^*^	1,081.93 ± 271.03	2,227.70 ± 422.83^*^
PDL+HEMO	7.00 ± 1.57	8.00 ± 1.12	5.00 ± 0.58	5.00 ± 0.77	16.00 ± 1.56	19.50 ± 2.35	797.21 ± 91.32	1,010.25 ± 129.32

In bold statistical differences, asterisks for positive statistical significance (^*^), minus for negative statistical significance (–).

### Immunocytochemistry studies of neurons in culture

**Figure 8:**
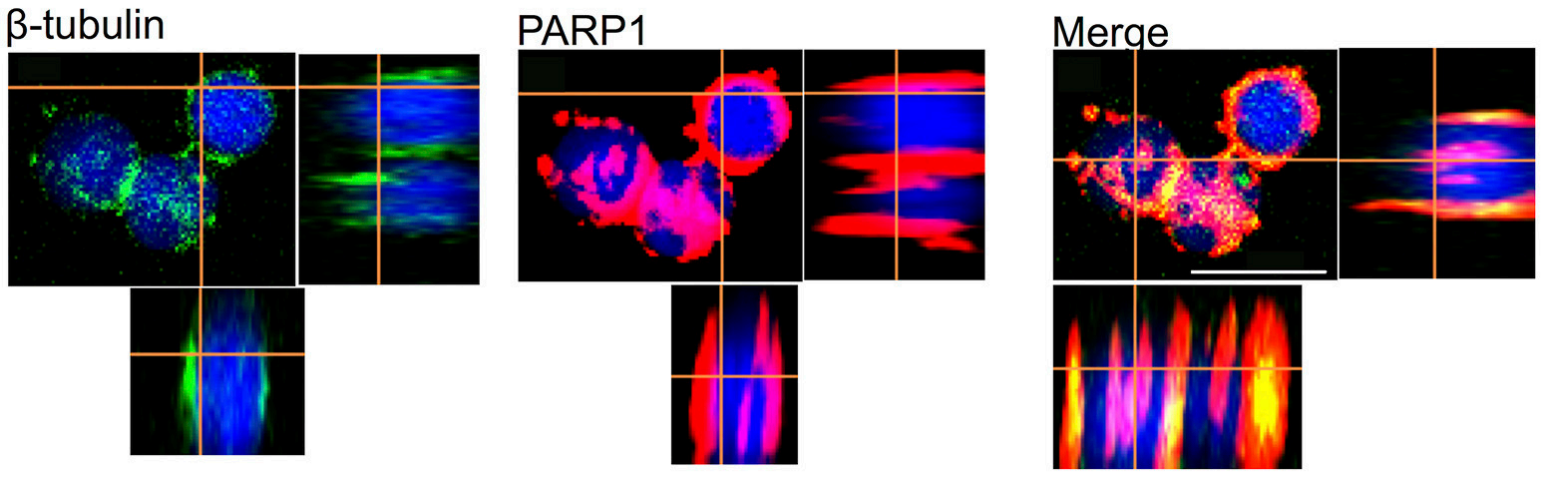
Merging of immunofluorescence images obtained from OL Neurons, β-Tubulin (Green), PARP1 (Red), nucleus (Blue). PARP1 and β-Tubulin are co-localized in cell cytosol, which exhibits yellow stain. Cytoplasmic PARP is also located in nuclei indicated by the overlap of red and blue, which displays in magenta; white scale bar indicates 10 μm.

Because neuronal survival and development depend critically on the presence of certain proteins such as the dynamic cytoskeletal proteins and enzymes, we next tested whether these neurons *in vitro* express such elements in their cell bodies and processes. Specifically, we tested the expression of the cytoskeletal protein β-tubulin and the enzyme poly ADP-ribose polymerase (PARP1, cytosol form). Our data showed that both β-tubulin and PARP1 were strongly expressed in all cultured OL neurons (Figure 8). The majority of PARP1 (red) was distributed in the cytosol of neurons and neuronal processes and a few magenta spot surrounding the nuclear area due to a weak cross-reactivity to PARP1 nuclear isoform. Interestingly, β-tubulin (green) is exclusively located in the neuronal cytosol and selectively co-localized with PARP1 in the soma-soma contact sites. In neurons that did not form contact with other cells, β-tubulin appeared to exhibit a non-localized distribution in the entire cytosol. These results are in agreement with our previously published results on the PARP1 immunohistochemistry in the central nervous system (CNS) of *Octopus vulgaris* (De Lisa et al., 2012a).

### Regeneration experiment on VSFS neurons

**Figure 9:**
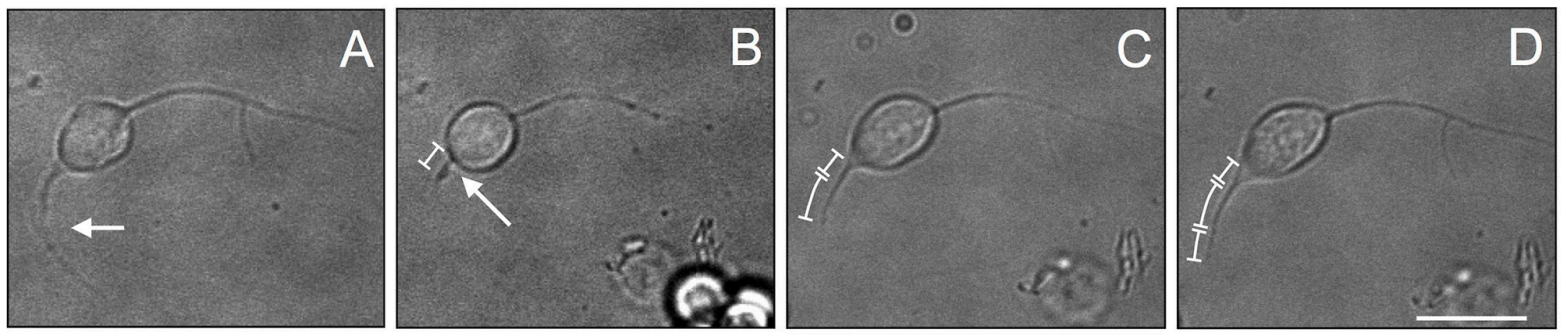
Re-growth of neurite after cut experiment in VSFS neuronal cell cultured in a PDL coated dishes with L15 enriched with FBS; Figures neurite growth in culture **(A)**, neurite after the axotomy **(B)**, neurite re-growth after 1 **(C)** and 2 **(D)** h; white scale bar indicates 10 μm.

In order to test the effectiveness of PDL substrate with 4% FBS to induce regeneration of injured axons, we performed a regeneration experiment on VSFS neurons. We first cultured neurons in the presence of FBS and allowed them to develop active growing processes. The selected long neurite was then severed by a sharp glass pipette mounted on a micromanipulator. Interestingly, we found that the axotomized axon regenerated its severed process within a short time period (Figure 9). Time-lapse phase contrast images revealed that neurons after axotomy exhibited a faster re-growth in the first hour (0–1 h) after cut (Figure 9C) and slightly slower rate in the second hour after axotomy (Figure 9D). These results thus underscore the regenerative capacity of octopus neurons at the level of single neurons and their axons, and demonstrate that the intrinsic regeneration capacity of axons is preserved in cell culture.

## Discussion

In the present study, we are the first to develop a protocol for primary culture of neurons from *Octopus vulgaris* VSFS and OL lobes. Previously, white body cells from *O. vulgaris* (Necco and Martin, 1963) and neurons from the stellate ganglion of *O. rubescens* (Gilly et al., 1997) were cultured.

Specifically, we examined the efficacy of commonly used enzymes (papain vs. collagenase/trypsin) for tissue dissociation, adhesive molecules (PLL vs. PDL) for dish coating, and supportive factors (HEMO vs. FBS) for promoting neuronal survival and growth. Our study showed that papain dissociation and PDL coating was a good combination for promoting cell yield and neuronal attachment. Adding growth permissive factors such as HEMO or FBS to the defined medium facilitated neuronal growth in culture, in which FBS was found to be more potent than HEMO in promoting neuritic branches and elongation. The octopus brain is characterized by highly heterogeneous population of neurons and other cell types, and the protocol developed here allowed us to have high cell culture efficiency for both VSFS and OL neurons.

In addition, we also provided interesting data showing that these neurons express cytoskeletal proteins like β-tubulin and enzyme PARP1. The latter is involved in octopus adult neurogenesis, differentiation, and synaptic plasticity after learning and sensory integration (De Lisa et al., 2012a; De Maio et al., 2013; Bertapelle et al., 2017). Lastly, we provided the first direct evidence that the axotomized octopus neurons exhibit robust regenerative ability *in vitro*.

Octopuses are one of the most intelligent invertebrates with an exceptional ability to sense environmental stimuli, to escape from predators, and to learn and execute new tasks (Mather, 1991, 2008; Kuba et al., 2006b, 2010; De Lisa et al., 2012a; Bertapelle et al., 2017). The octopus is phylogenetically remote from vertebrates, and much more sophisticated in its behavior than other mollusc, making it an ideal model for a comparative analysis of brain mechanisms selected during evolution for the mediation of complex behaviors. In addition, cephalopods are protected animals so the development of neural cell culture could be considered as an “alternative method” to study selected lobes of central brain, such as VSFS and OL which are involved in memory and learning and in the integration of sensory stimuli, as well as in adult neurogenesis (Bertapelle et al., 2017).

In molluscs such as the *Aplysia californica* and *Lymnaea stagnalis*, the central nervous system is arranged into a ring of 10–11 ganglia, which comprises about 20,000 large neurons (soma diameters in the range of 20–150 μm) and are wrapped with visible outer and inner sheath. In contrast, the octopus nervous system is organized into three parts, controlling specific functions: (1) the highly developed central nervous system (CNS), which wraps around the esophagus, is situated inside a cartilaginous capsule and composed of ~45 million neurons, packed in fused ganglia, forming a supraoesophageal and suboesophageal masses; (2) the two optic lobes, which are connected with CNS by a distinct optic traits (on which the olfactory lobes and optic glands are localized), contain ~180 million neurons; and (3) the Peripheral Nervous System (PNS) of the arms (and body) which is composed of ~350 million neurons (Young, 1971; Budelmann, 1995). The soma diameters of octopus central neurons are in the range of 5–20 μm (Young, 1971), which are comparable to mammalian central neurons and are much smaller than mollusc neurons. Because of the above similarities with mammalian brain tissue, we adopted a similar dissociation enzyme approach for octopus neurons.

To test the optimal dissociation enzymes, we evaluate the effects of two commonly used dissociation enzymes in mammalian cell culture system (papain vs. collagenase) on octopus cell dissociation efficiency, cell adhesion, survival, and neurite growth. We discovered that papain and collagenase both were potent in breaking the connective tissue of octopus brain, although neurons treated with papain enzyme attached better in culture. These data indicate that the connective tissue for maintaining the structural integrity of octopus neurons is more comparable to mammalian brain tissue. Our study is thus in agreement with several other pioneering studies showing that papain dissociation provides higher yields of viable, morphologically intact neurons than other proteases such as collagenase and/or trypsin in a variety of other cell culture system (Huettner and Baughman, 1986; Finkbeiner and Stevens, 1988; Dreyfus and Black, 1990). However, we observed a difference in the effect of coating reagents (PLL and PDL) in cell adhesion and growth in papain treated neurons. We found that neurons cultured on PLL-coated glass coverslips attached better and grew well—even in the absence of endogenous and exogenous growth factors (Figure 2 and Table 1). Cell cultured on PDL coated surface had a lower density of attached cells and their growth required the addition of HEMO and FBS, in which FBS had a significant effect on promoting neural growth than HEMO (Figures 2, 7). Although both polymers of PLL and PDL provided positive charges to the glass surface for promoting cell attachment, PLL papered to be a more favorable coating agent for culturing neurons from invertebrates (Syed et al., 1993; Saver et al., 1999), while PDL is more commonly used for vertebrate cell culture surfaces (Xu et al., 2010; Getz et al., 2016). It is important to note that concanavalin A (ConA) was used in the cell culture of giant fiber lobe neurons of the squid (Gilly et al., 1990). Previous studies in cell cultures of *Lymnaea* and *Hirudo medicinalis* neurons have, however, demonstrated that neurons grown on ConA substrates tend to primarily form improper electrical, but not the proper chemical synapses (Syed et al., 1999). Nevertheless, it is very interesting to test the effects of ConA pre-coating on the extent of neurite outgrowth of Octopus neurons in our future experiments.

All invertebrate neuronal cell cultures require either hemolymph as demonstrated in Aplysia (Schacher and Proshansky, 1983; Ghirardi et al., 1996; Schmold and Syed, 2012) or brain conditioned medium as in *Lymnea* (Ridgway et al., 1991; Munno et al., 2000) or *Helisoma* (Cohan et al., 2003), which is not feasible with *O. vulgaris*, we sought to examine the effects of growth factors such as HEMO and FBS (Figures 5, 6) on cell survival and neural growth after day 1 and 4 in culture. On day 1, the survival of VSFS neurons dissociated with papain enzyme treatment was better on the PLL substrate when HEMO was added. The neurons typically grew extensive neurites and exhibited complex branching patterns (Figures 4E,L), probably due to the presence of some unknown endogenous growth factor, which may have accompanied the cells during the dissociation (Hyland et al., 2014).

Nevertheless, after 4 days, this growth-promotion stopped and an inversion of the trend took place (Figures 4F,M)—suggesting that the neurons may require extrinsic factors for their continued growth/viability. On the contrary, over a culture period of 4 days VSFS neurons, plated on PDL+FBS, responded positively to all selected parameters (Table 2). In the same way, OL neurons cultured with PDL+FBS for 4 days provided efficient and satisfying results, inducing cell growth.

These results suggest that the trophic factors present in the blood of these animals may likely be evolutionary conserved across vertebrate and invertebrate species (Ridgway et al., 1991; Munno et al., 2000; Castellanos-Martinez et al., 2014).

*Octopus vulgaris* has a closed vascular system and a CNS expressing a larger number of proteins involved in specific junctions, transporters and enzymes, than observed in other mollusc model organisms, such as *A. californica*, characterized by an open vascular system(Zhang et al., 2012). Moreover, *Octopus vulgaris* has specific gene expression indicative of a vertebrate-like Blood-Brain-Barrier (Zhang et al., 2012). This issue is supported by the relatively abundant amount of the Acetylcholinesterase (ACHE) in the FBS (Doctor et al., 1990). Recently ACHE, well known for its key role in synaptic and extrasynaptic locations in the nervous system, has been cloned and characterized in *O. vulgaris* (Fossati et al., 2015). The *O. vulgaris* ACHE sequence showed 43–46% identity with vertebrate ACHEs and 43–47% with invertebrate deuterostomes ACHEs and possesses main catalytic and non-catalytic functional sites (Fossati et al., 2015). It will be very interesting to test whether the presence of ACHE in the FBS plays any role in cell survival and growth in our culture in the future.

These data indicated that *Octopus vulgaris* neuronal surface proteins may share some similarity with vertebrate neurons (Cardone and Roots, 1990; Phan et al., 2016), as happens for Protocadherins gene families, involved in neuronal process outgrowth and prevent neurite self-entanglement, that in *O. bimaculoide*s shared features with the mammalian clustered Protocadherins (Liscovitch-Brauer et al., 2017; Wang and Ragsdale, 2017).

In the present study, we have demonstrated that FBS neurotrophic effects improve cell culture in both VSFS and OL neurons. When neurons were cultured for 4 days in PDL coating with the presence of FBS, many of them formed physical contacts with adjacent cells either in soma-soma, soma-neurites and neurite-neurite configurations. To study further these connections, we performed an immunofluorescence experiment on FBS cultured OL neurons using the antibodies against the cytoskeletal protein, β-tubulin, and the PARP1, an enzyme was previously localized in OL and VSFS of *O. vulgaris* CNS, involved in neuronal plasticity (De Lisa et al., 2012a; De Maio et al., 2013; Bertapelle et al., 2017). As expected, the immunoreactivity of both antibodies was widespread throughout the cytoplasm of the cell somata and only few magenta spots were visible in the nuclei due to a weak cross-reactivity to PARP nuclear isoform. Interestingly, the cytoskeletal protein and PARP enzyme tend to be co-localized (spot yellow) in the cell contact sites between two contacted neurons (Figure 8), indicating that these sites were likely undergoing active assembly and functional development. A functional evidence for potential synaptic contact between cells however awaits further electrophysiological experimentation.

In summary, the results highlighted here demonstrate that an exogenous factor (FBS) improved cell survival, neural growth, and probably network formation in a more effective manner than an endogenous factor (HEMO). We hypothesize that a growth factor/s present in HEMO provide an initial push along with other factors, such as neurosteroids (De Lisa et al., 2012b), working synergistically in promoting neuronal growth. In support of this notion, studies have shown that the expression of oestradiol receptor in the lobes of *O. vulgaris* CNS has been recently associated neural plasticity (De Lisa et al., 2012b). The effectiveness of VSFS culture conditions (PDL+FBS) was supported by the re-growth of an injured neuron as demonstrated in the regeneration experiment. This result could open a new scenario in which the CNS of *O. vulgaris*, characterized by the presence of nervous districts comparable to vertebrates' specific areas, could be a useful model to study the neurites regeneration after injury (Koert et al., 2001; Lee and Syed, 2004; Onizuka et al., 2005, 2012; Walters and Moroz, 2009).

## Author contributions

The authors have made the following declarations about their contributions: Conceived and designed the experiments: VM, FX, NS, and AD. Performed the experiments: VM and FX. Analyzed the data: VM, FX, NS, and AD. Contributed reagents, materials, analysis tools: AD. Wrote the paper: VM and AD. Revised the manuscript: VM, FX, NS, GP, and AD.

### Conflict of interest statement

The handling Editor declared a shared affiliation, though no other collaboration, with the authors VM, GP, and AD. The other authors declare that the research was conducted in the absence of any commercial or financial relationships that could be construed as a potential conflict of interest.

## Supplementary material

Figure S1 Different cell types from selected lobes (VSFS and OL): neurons from medulla of the OL **(A)**; glial cells from plexiform zone of the OL **(B)**; glial cell (1), large cell (2) and amacrine cell (3) from Vertical lobe **(C)**; amacrine cell (4), and bipolar neuron (5) from frontal system lobe **(D)**; white scale bar indicates 10 μm.

## Catalog numbers for all chemicals:

Anti-β III Tubulin antibody, Cat. No. ab78078, abcam;

Collagenase P, Cat. No. 11 213 857 001, Sigma Aldrich, St. Louis, MO, USA;

DAPI, Cat. No. D9542, Sigma Aldrich, St. Louis, MO, USA;

Fetal Bovine Serum, Cat. No. F4135 Sigma Aldrich, St. Louis, MO, USA;

FITC-conjugated goat anti-mouse IgG, Cat. No. 62-6511, Thermo Fisher Scientific;

Glycerol, Cat. No. G2025, Sigma Aldrich, St. Louis, MO, USA;

Goat Serum, Cat. No. G6767, Sigma Aldrich, St. Louis, MO, USA;

HEPES, Cat. No. H4034 Sigma Aldrich, St. Louis, MO, USA;

KCl, Cat. No. P5405 Sigma Aldrich, St. Louis, MO, USA;

Leibovitz's L-15 Medium, Cat. No. 11415064, ThermoFisher Scientific, Waltham, MA, USA;

Papain, Cat. No. P4762 Sigma Aldrich, St. Louis, MO, USA;

Paraformaldehyde, Cat. No. 158127, Sigma Aldrich, St. Louis, MO, USA; Penicillin-Streptomycin solution, Cat. No. P4333, Sigma Aldrich, St. Louis, MO, USA; Phenol red solution, Cat. No. P0290 Sigma Aldrich, St. Louis, MO, USA;

Phosphate Buffered Saline, Cat. No. P5493, Sigma Aldrich, St. Louis, MO, USA;

Poly-D-lysine solution, Cat. No. A-003-M Sigma Aldrich, St. Louis, MO, USA;

Poly-L-lysine solution, Cat. No. P4707 Sigma Aldrich, St. Louis, MO, USA;

polyclonal poli (ADP-ribose) polymerase (PARP1) primary antibody, Cat. No. sc-8007, Santa Cruz Biotechnology Inc;

Rhodamine conjugated goat anti-rabbit IgG, Cat. No. 31670, Thermo Fisher Scientific;

Sodium chloride, Cat. No. S5886 Sigma Aldrich, St. Louis, MO, USA;

Triton-X 100, Cat. No. T8787, Sigma Aldrich, St. Louis, MO, USA;

Trypan Blue solution, Cat. No. T8154, Sigma Aldrich, St. Louis, MO, USA;

Trypsin, Cat. No. T2601 Sigma Aldrich, St. Louis, MO, USA.

## Abbreviations

DAPI: 4′,6-Diamidino-2-phenylindole dihydrochloride
FBS: Fetal Bovine Serum
HEMO: Hemolymph
L15-ASW: L15-supplemented artificial sea water
OL: optic lobes
PARP1: poli (ADP-ribose) polymerase
PBS: Phosphate Buffered Saline
PDL: Poly - D - lysine
Pen/Strep: Penicillin-Streptomycin solution
PLL: Poly – L - lysine
VSFS: vertical-superior frontal system mass.
