# Corrigendum: A Novel Approach to Primary Cell Culture for *Octopus Vulgaris* Neurons

**DOI:** 10.3389/fphys.2018.01900

**Published:** 2019-01-10

**Authors:** Valeria Maselli, Fenglian Xu, Naweed I. Syed, Gianluca Polese, Anna Di Cosmo

**Affiliations:** ^1^Department of Biology, University of Naples Federico II, Napoli, Italy; ^2^Department of Biology, Saint Louis University, Saint Louis, MO, United States; ^3^Department of Cell Biology and Anatomy, Cumming School of Medicine, University of Calgary, Calgary, AB, Canada

**Keywords:** primary neuron cell culture, *Octopus vulgaris*, cephalopods, marine invertebrates, central nervous system, vertical-superior frontal system, optic lobes, axon regeneration

In the original article, there was a mistake in Figure 6 as published. There was an unintentional error in the table composition of Figure 6C. The corrected Figure 6 appears below.

**Figure 6:**
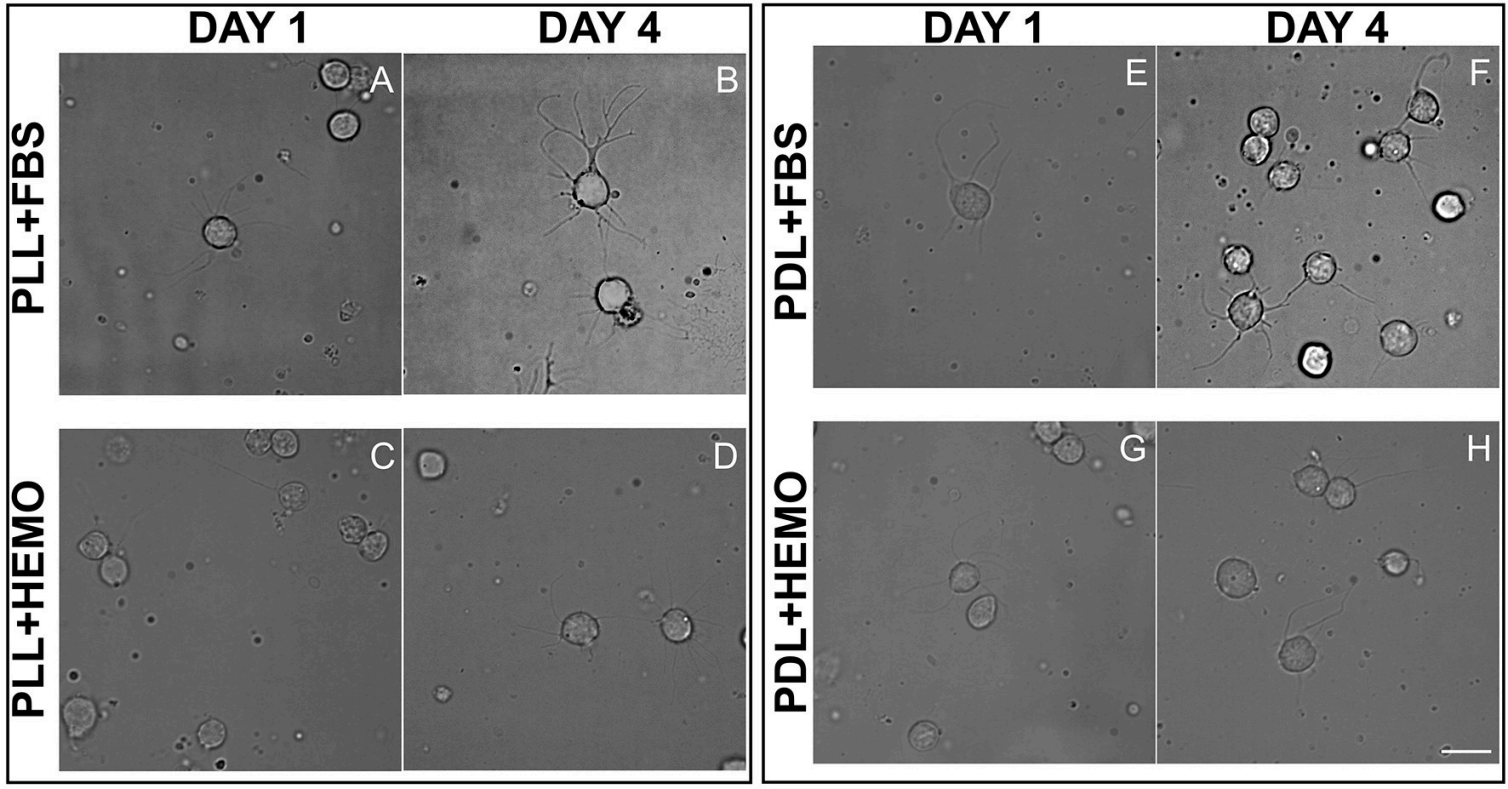
Cells from OL plated on PLL **(A–D)** and PDL **(E–H)** and coated dishes, cultured for 4 days in L15 medium with the addition of FBS **(A,B,E,F)** or HEMO **(C,D,G,H)**; white scale bar indicates 10 μm.

The authors apologize for this error and state that this does not change the scientific conclusions of the article in any way. The original article has been updated.

## Conflict of Interest Statement

The authors declare that the research was conducted in the absence of any commercial or financial relationships that could be construed as a potential conflict of interest.

