# Supplementary figures and images for: A Novel Approach to Primary Cell Culture for Octopus vulgaris Neurons
Source: Front Physiol. 2018 Apr 3;9:220. doi: 10.3389/fphys.2018.00220 (PMC5891582; doi:10.3389/fphys.2018.00220)

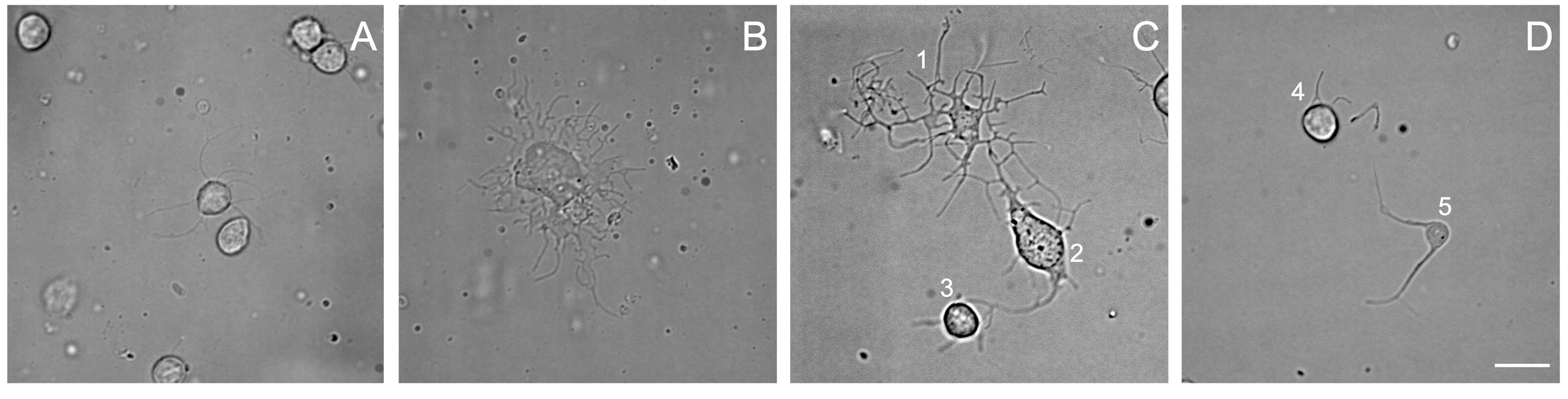

Supplement: Figure S1 — Different cell types from selected lobes (VSFS and OL): neurons from medulla of the OL (A); glial cells from plexiform zone of the OL (B); glial cell (1), large cell (2) and amacrine cell (3) from Vertical lobe (C); amacrine cell (4), and bipolar neuron (5) from frontal system lobe (D); white scale bar indicates 10 μm. [file Image1.TIFF]
